# Cognitive-Locomotor Dual-Task Interference in Stroke Survivors and the Influence of the Tasks: A Systematic Review

**DOI:** 10.3389/fneur.2020.00882

**Published:** 2020-08-18

**Authors:** Anne Deblock-Bellamy, Anouk Lamontagne, Andréanne K. Blanchette

**Affiliations:** ^1^Faculty of Medicine, Universite Laval, Quebec City, QC, Canada; ^2^Center for Interdisciplinary Research in Rehabilitation and Social Integration (Cirris) – CIUSSS de la Capitale-Nationale, Quebec City, QC, Canada; ^3^School of Physical and Occupational Therapy, McGill University, Montreal, QC, Canada; ^4^Centre for Interdisciplinary Research in Rehabilitation of Greater Montreal (CRIR), Jewish Rehabilitation Hospital - CISSS de Laval, Laval, QC, Canada; ^5^Department of Rehabilitation, Universite Laval, Quebec City, QC, Canada

**Keywords:** stroke, dual-task, interference, locomotion, cognition

## Abstract

**Background:** Walking in the community can be challenging for stroke survivors. The fact that community walking often requires performing another task while walking further adds to this challenge and can lead to a deterioration of performance in one or both tasks.

**Objective:** To review the existing literature about cognitive-locomotor dual-task interference (DTI) magnitude and pattern while walking in patients with stroke and to explore the influence of tasks' nature on DTI. Moreover, this review investigated the differences in DTI between stroke survivors and age-matched healthy adults.

**Methods:** The literature search was conducted in 4 databases (MEDLINE, CINAHL, EMBASE and PEDro). Two authors independently identified relevant studies based on predetermined selection criteria. Among these criteria, studies had to include both locomotor and cognitive DTI. Methodological quality of the studies was independently assessed by two raters using a standardized checklist. Studies were categorized according to the nature of the locomotor and the cognitive tasks.

**Results:** A total of twenty studies, with good to high methodological quality, were selected. Task combinations, outcome measures and participants characteristics varied widely from one study to another. Despite heterogeneous results across studies, mutual DTI (decrements in both locomotor and cognitive performance) was the most frequently observed pattern in participants with stroke. Interestingly, this DTI pattern was systematically obtained when participants had to avoid obstacles while walking. DTI seemed also to be influenced by the nature of the cognitive task. Compared to age-matched healthy participants, stroke survivors had greater DTI. Mutual interferences were also more frequently observed in stroke survivors than in age-matched healthy adults.

**Conclusions:** DTI magnitude and pattern in persons with stroke varied considerably across studies. Multiple factors, including nature of the tasks, may influence dual-task abilities when assessing individuals with stroke. Consequently, dual-task assessments should be performed in similar contexts of individuals' daily lives to ensure ecological validity.

## Introduction

The ability to get out and about community is considered essential or very important by more than 70% of stroke survivors (1). While 80% of stroke rehabilitation inpatients can walk indoors, only 27% are able to perform 4 of the essential skills needed to walk independently in the community, including independence with stairs, ability to negotiate inclined surface, walking at a minimal speed of 0.8 m/s and walking on a distance of 367 meters or higher in 6 min (2). Sensorimotor and perceptual impairments which are common following stroke (3–5) can have a detrimental impact on community ambulation (6–8). Cognitive functions, such as divided attention, are also crucial to walk independently and safely in the community (9). In everyday living, people have frequently to walk while performing another task such as discussing with someone, texting or recalling a shopping list. This ability to perform two tasks simultaneously can be assessed using a dual-task paradigm. When using this approach, dual-task performance is compared with single-task performance, with only one task executed at a time in the latter condition. Several studies have demonstrated that adding a cognitive task while walking may deteriorate the performance of either or both tasks compared with the performance of each task executed separately (10, 11). Cognitive-locomotor dual-task interference (DTI) was previously documented in populations with and without locomotor or cognitive deficits, at all ages (12). Different theories suggest that DTI may be the result of limited attentional resources, but the latter may reflect a broad variety of underlying mechanisms or processes. One of these frameworks, referred to as *Central Capacity Sharing* (13), proposes that both tasks share available processing resources. These resources are limited, however. Thus, tasks might be performed in parallel, with decrement in performances when resources are overloaded. Another theoretical framework, the *Bottleneck Theory*, rather proposes that processing involved in each task may need simultaneous access to processor that can only act with one input at the time (14). Consequently, the processing of the second task might be postponed.

Several studies have already demonstrated that neurological lesions, as well as age-related sensorimotor decline, may compromise the ability to perform a cognitive task while walking (15–18). A previous meta-analysis reviewed the literature, published until the end of 2009, on the effects of adding a concurrent cognitive task on gait performance (15). In this study, a subgroup analysis demonstrated greater locomotor DTI in individuals with neurological disorders than in healthy older adults. However, DTI in cognitive performance was not reported in this review. Given the possibility of attentional prioritization (19, 20) or asymmetrical resources allocation between both tasks, it is crucial to characterize both motor and cognitive performance, in single- and dual-task conditions, to accurately calculate and interpret DTI. Otherwise, conclusions that can be drawn about DTI pattern remain limited and potentially incorrect. A scoping review, published in 2013, focuses exclusively on research studies that measured both locomotor and cognitive interference in stroke survivors (10). Overall, this review suggests that stroke survivors are likely to demonstrate significant decrements in locomotor performance only or in both locomotor and cognitive performances. However, none of the studies included in this review have exposed stroke survivors to complex locomotor task, such as walking while negotiating obstacles, which therefore limits the scope of the results about the potential impact of cognitive-locomotor interference in their daily lives in the community. Moreover, no conclusion about the specific impact of the lesion on DTI can be derived from this review, since their results were not compared with age-matched healthy participants.

In order to fill the gaps in the literature, the aims of the present systematic review were therefore (1) to examine cognitive-locomotor interference magnitude and pattern while dual-tasking in patients with stroke; (2) to explore the influence of the nature of the tasks on DTI, and (3) to investigate the differences in DTI magnitude and pattern between stroke survivors and age-matched healthy adults.

## Methods

This systematic review was completed according to the Preferred Reporting Items for Systematic Review and Meta-analyses (21).

### Search Strategy

A systematic literature search was conducted using the following electronic databases: MEDLINE via PubMed, CINAHL, EMBASE, and PEDro. A search strategy combining keywords and indexed vocabulary related to three key concepts (stroke; gait; dual-task) was used (detailed search strategy in Supplementary Material). Indexed vocabulary was adapted for each database. Databases were searched from inception through January 31st, 2019.

### Selection of Studies

After deleting duplicates, the titles and abstracts were screened by two independent reviewers (AD-B and AB). Relevant full texts were then examined to determine their eligibility for inclusion in the review. In case of disagreement, a third reviewer had been consulted. Articles were included if they met the following inclusion criteria: (1) assessment of dual-task ability while performing a locomotor and a cognitive task simultaneously, (2) study participants were adults with stroke, (3) locomotor AND cognitive DTI were reported as outcomes, (4) original scientific article written in English or French. In order to reach the third objective specifically, studies including a comparison group of healthy age-matched adults were selected. Case series, case reports, conference proceedings and abstracts, letters to the editor, opinion papers, theses, reviews and meta-analyses were not considered for this review.

### Methodological Quality Assessment

The *Standard Quality Assessment Criteria for Evaluating Primary Research Papers* checklist, developed by Kmet et al. (22), was used to assess the methodological quality of each included study. This checklist assesses 14 items including study objectives and design, participants' recruitment and description, sample size, outcomes measures, data analysis, results and conclusions. Two raters [AD-B and AB] independently evaluated each article. In the absence of a consensus, discrepancies were discussed, and a third reviewer [AL] was consulted for final decision. Studies were then categorized based on the following methodological quality index: “high quality” for scores >80%, “good quality” for scores between 80 and 70%, “moderate quality” for scores between 69 and 50% and “low quality” for scores lower than 50% (23).

### Data Extraction

Relevant data were extracted by one reviewer (AD-B) and a second reviewer (AB) checked the accuracy of extraction. Extracted data included information on study design, participants' characteristics (sample size, gender, age, delay after stroke, baseline locomotor, and cognitive function), locomotion and cognitive tasks performed, outcome measures, and main results.

### Data Analyses

For the analyses, studies were clustered according to the nature of the locomotor and the cognitive tasks. Locomotor tasks consisted of: (1) simple forward walking, (2) walking with direction changes (oval or circle walking, walking back and forth with 180-degree turns, Timed Up and Go Test [TUG]), (3) walking with obstacles (crossing or circumventing), and (4) other challenging locomotor tasks (walking paths combining obstacles, tandem-walking and stepping onto targets). Cognitive tasks were categorized according to the main mental processes required to execute them, as determined in Al-Yahya et al. (15): (1) mental tracking tasks, (2) discrimination and decision-making tasks, (3) verbal fluency tasks, (4) working memory tasks, and (5) reaction time tasks.

For each study, DTI pattern was characterized, based on the framework proposed in Plummer et al. (10), i.e., deterioration in only one of both performances (*cognitive-related motor interference* or *motor-related cognitive interference*), deterioration in both performances (*mutual interference*), or no performance change (*no dual-task interference*).

To examine cognitive-locomotor DTI in individuals with stroke and explore the influence of the nature of the tasks (Objectives 1 and 2), studies including statistical comparisons between single- and dual-task performances (locomotor and cognitive) were considered. In order to compare the magnitude of DTI between stroke and healthy individuals (Objective 3), each study performing (1) *t*-tests between DTI (locomotor and cognitive) of stroke and healthy individuals or (2) two-way ANOVA with group^*^task interaction was considered. Significant *t*-tests results or group^*^task interactions indicated a difference between stroke and healthy individuals. Moreover, DTI between groups was also considered different when a significant difference was reported by the authors, even if statistical results were not shown.

## Results

### Selection of Articles

**Figure 1:**
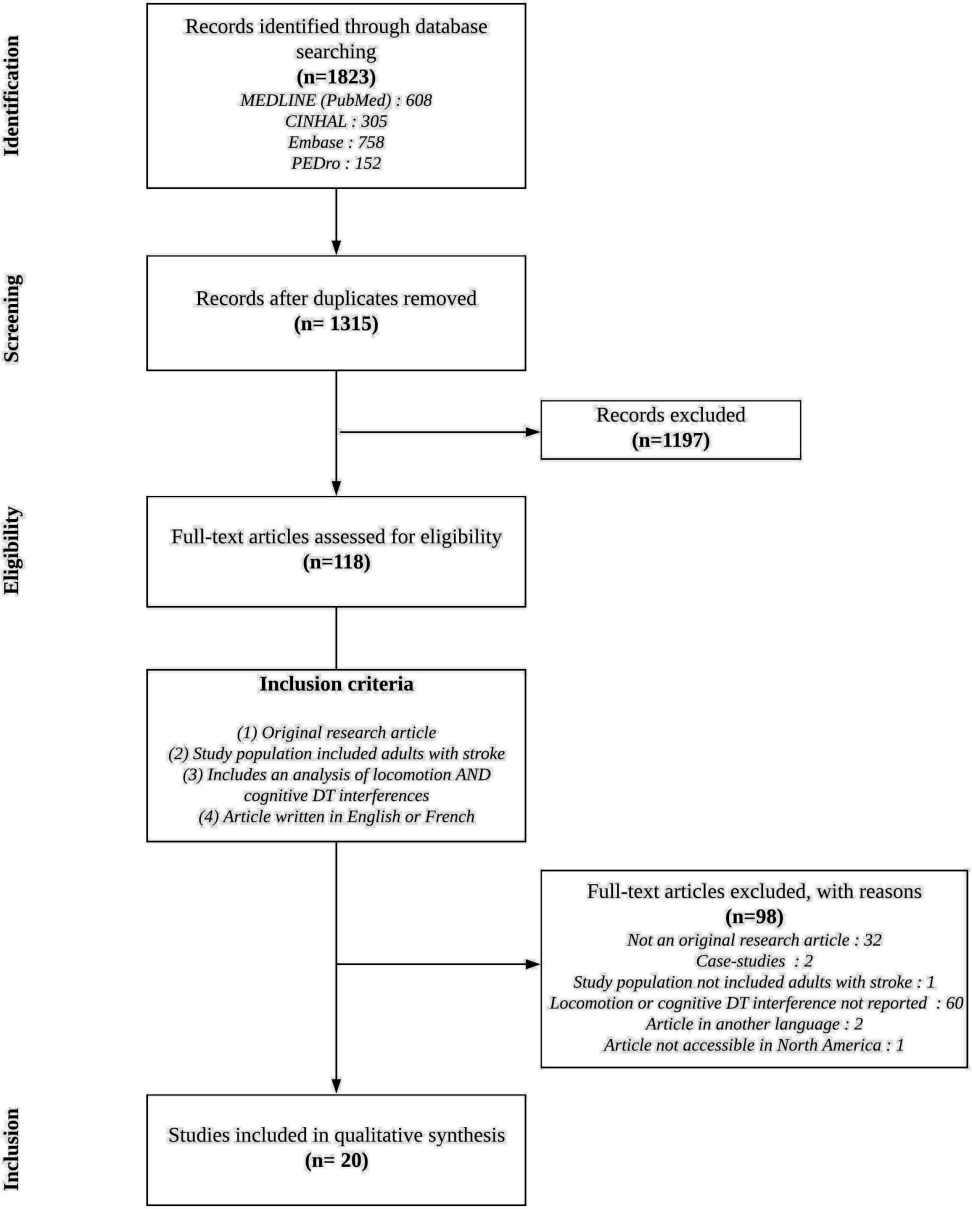
Flow diagram of the study selection process.

The literature search resulted in the identification of 1,823 references from which 508 duplicates were removed. The remaining 1,315 references had their titles and abstracts screened for eligibility. One hundred and eighteen (118) full texts were read, and 98 articles did not meet the inclusion criteria. Twenty articles were therefore included in this systematic review Figure 1; (20, 24–42). Among these, 17 studies were considered for the first and second objectives, while 7 studies contributed to the third objective.

### Methodological Quality

All except one study (38) had a cross-sectional design. The methodological quality scores ranged from 70 to 100% (mean ± standard deviation: 86.1% ± 8.8; Table 1). Methodological quality of 12 studies was considered as high (>80%), while 8 had a good methodological quality (between 70 and 80%).

**Table 1:** Methodological quality assessment of included studies.

**References**	**Items**														**Score (%)**
	**1**	**2**	**3**	**4**	**5**	**6**	**7**	**8**	**9**	**10**	**11**	**12**	**13**	**14**	
Al-Yahya et al. (25)	Y	Y	P	Y	n/a	n/a	n/a	Y	Y	P	Y	n/a	Y	P	85
Aravind and Lamontagne (39)	Y	Y	Y	Y	n/a	n/a	n/a	Y	Y	Y	Y	n/a	Y	Y	100
Chan and Tsang (26)	P	Y	P	Y	n/a	n/a	n/a	Y	Y	Y	Y	n/a	Y	Y	90
Denneman et al. (42)	Y	Y	Y	Y	n/a	n/a	n/a	P	Y	Y	Y	n/a	Y	Y	95
Dennis et al. (27)	Y	Y	P	Y	n/a	n/a	n/a	P	P	Y	P	n/a	Y	P	75
Feld et al. (40)	Y	Y	P	P	n/a	n/a	n/a	P	Y	P	Y	n/a	Y	Y	80
Goh et al. (35)	Y	Y	Y	P	n/a	n/a	n/a	Y	Y	Y	Y	n/a	Y	Y	95
Hyndman et al. (28)	Y	Y	P	P	n/a	n/a	n/a	P	Y	P	Y	n/a	Y	Y	80
Kemper et al. (29)	P	Y	P	P	n/a	n/a	n/a	P	P	Y	P	n/a	Y	Y	70
Manaf et al. (30)	Y	Y	P	Y	n/a	n/a	n/a	Y	Y	Y	Y	n/a	Y	Y	95
Mori et al. (24)	Y	Y	P	Y	n/a	n/a	n/a	Y	P	P	Y	n/a	Y	P	80
Pang et al. (38)	Y	Y	Y	P	Y	Y	n/a	Y	Y	Y	Y	Y	Y	Y	96
Patel and Bhatt (31)	Y	Y	P	P	n/a	n/a	n/a	Y	Y	P	P	n/a	Y	Y	80
Plummer-D'Amato et al. (41)	P	Y	P	Y	n/a	n/a	n/a	Y	Y	Y	Y	n/a	Y	P	85
Pohl et al. (32)	Y	Y	Y	Y	n/a	n/a	n/a	Y	Y	Y	Y	n/a	Y	P	95
Regnaux et al. (33)	Y	Y	P	P	n/a	n/a	n/a	P	P	P	Y	n/a	Y	P	70
Timmermans et al. (20)	Y	Y	P	Y	n/a	n/a	n/a	P	Y	Y	Y	n/a	Y	Y	90
Tisserand et al. (34)	Y	Y	P	Y	n/a	n/a	n/a	Y	P	Y	Y	n/a	Y	Y	90
Yang et al. (37)	Y	P	Y	Y	n/a	n/a	n/a	Y	Y	Y	Y	n/a	Y	P	90
Yang et al. (36)	Y	Y	P	Y	n/a	n/a	n/a	Y	Y	P	P	n/a	Y	P	80
Mean (± SD)															86.1 ± 8.8

Studies presented in alphabetic order. Y, yes (2 points); P, partial (1 point); N, no (0 points); n/a, not applicable; SD, standard deviation. Item numbers on the Standard Quality Assessment Criteria for Evaluating Primary Research Paper are as follows: (1) Question/objective sufficiently described? (2) Study design evident and appropriate? (3) Method of subject/comparison group selection or source of information/input variables described and appropriate? (4) Subject (and comparison group, if applicable) characteristics sufficiently described? (5) If interventional and random allocation was possible, was it described? (6) If interventional and blinding of investigators was possible, was it reported? (7) If interventional and blinding of subjects was possible, was it reported? (8) Outcome and (if applicable) exposure measure(s) well-defined and robust to measurement/misclassification bias? Means of assessment reported? (9) Sample size appropriate? (10) Analytic methods described/justified and appropriate? (11) Some estimate of variance is reported for the main results? (12) Controlled for confounding? (13) Results reported in sufficient detail? (14) Conclusions supported by the results?

### Description of Included Studies

#### Participants' Characteristics

A total of 671 participants with a stroke were recruited in the included studies. While participants in the chronic phase of recovery (at least 6 months after the stroke) were exclusively recruited in 15 studies (24–38), both subacute (at least 1 month after the stroke) and chronic stroke participants were included in 4 studies (20, 39–41). The remaining study excluded chronic participants (42). Participants' mean age ranged from 46.6 ± 12.3 to 77.2 ± 5.8 years old. Sixteen studies provided information about participants' gender. In these studies, the proportion of women recruited ranged between 10.5 and 50.8 %. All studies except two, have reported indicators of participants' cognitive function. The mean scores reported in each study suggested that most participants had no cognitive impairment. All included participants were able to walk independently on short distances. Among the 17 studies reporting participants' walking velocity, means ranged between 0.5 and 1.1 m/s (Table 2).

**Table 2:** Participants' characteristics.

**References**	**Participants' group**	**Number of participants *n (female)***	**Age (years old)**	**Delay after stroke**	**Baseline locomotor function**	**Baseline cognitive function**
Al-Yahya et al. (25)	Stroke	19 *(2)*	59.6 ± 15.0	26.5 ± 27.5 months	0.5 ± 0.3 m/s	SOMCT: 25.4 ± 3.2
	Control	20 *(8)*	54.4 ± 9.4	–	1.0 ± 0.03 m/s	*NM*
Aravind and Lamontagne (39)	Stroke VSN +	13 *(NM)*	59.8 ± 7.7	10.5 ± 4.6 months	0.7 ± 0.2 m/s	MoCA: 26.1 ± 1.5
	Stroke VSN –	13 *(NM)*	60.8 ± 6.5	11.8 ± 1.6 months		MoCA: 27.1 ± 1.6
Chan and Tsang (26)	Stroke	59 *(30)*	62.4 ± 6.8	5.4 ± 4.8 years	TUG: 18.0 ± 8.9 s	MMSE: 27.9 ± 2.2
	Control	45 *(36)*	61.3 ± 4.8	–	TUG: 6.3 ± 1.0 s	MMSE: 29.4 ± 1.1
Denneman et al. (42)	Stroke	78 (*29*)	59.1 ± 10.8	31.9 ± 19.7 days	10MWT: 15.1 ± 8.8 s	DSST: 45.5 ± 18.1
Dennis et al. (27)	Stroke	21 *(8)*	61.0 ± 12.0	7–50 months	0.8 ± 0.4 m/s	RBANS: 83.3 ± 11.8
	Control	10 *(2)*	60.0 ± 6.0	–	1.4 ± 0.2 m/s	*NM*
Feld et al. (40)	Stroke	28 *(9)*	58.2 ± 16.6	8.9 months (median) (range: 3.7–19.4 months)	6MWT: 312.6 ± 133.6 m	MoCA: 26 (median) (range: 24.0–27.0)
Goh et al. (35)	Stroke	30 *(8)*	61.0 ± 5.7	87.3 ± 47.5 months	10MWT: 0.8 ± 0.3 m/s	Abbreviated mental test: <7
Hyndman et al. (28)	Stroke	36 *(15)*	66.5 ± 11.8	16.3 ± 11.8 months	0.7 ± 0.3 m/s	Star cancellation test (neglect): 52.9 ± 3.9
	Control	24 *(10)*	62.3 ± 11.6	–	0.9 ± 0.1 m/s	*NM*
Kemper et al. (29)	Stroke	10 *(NM)*	77.2 ± 5.8	24–36 months	“No walking deficits”	SPMSQ: 8.9 ± 1.1
	Control	10 *(NM)*	76.3 ± 5.4	–	“No walking deficits”	SPMSQ: 9.5 ± 0.9
Manaf et al. (30)	Stroke	10 *(3)*	49.3 ± 8.7	17.3 ± 7.6 months	TUG: 0.5 ± 1.8 m/s	MMSE: 28.7 ± 1.6
	Control	10 *(5)*	52.8 ± 5.4	–	*NM*	MMSE: 29.5 ± 1.6
Mori et al. (24)	Stroke	14 *(2)*	61.1 ± 9.3	> 6 months	10MWT: 0.9 ± 0.2 m/s	MMSE: 29.2 ± 1.1
	Control	14 (3)	66.3 ± 13.3	–	10MWT: 1.2 ± 0.2 m/s	MMSE: 28.7 ± 1.8
Pang et al. (38)	Stroke	84 (24)	61.2 ± 6.4	75.3 ± 64.9 months	Mild to moderate impairment in lower limb (CMSA)	MoCA: 26.0 ± 2.8
Patel and Bhatt (31)	Stroke	10 *(NM)*	56.8 ± 6.0	4.6 ± 2.6 years	10MWT: 9.0 ± 2.2 sec	SOCMT: 25.4 ± 2.6
	Control (no age-matched)	10 *(NM)*	25.6 ± 5.2	–	*NM*	*NM*
Plummer-D'Amato et al. (41)	Stroke	13 *(2)*	60.5 ± 15.3	8.7 ± 4.8 months	10MWT: 0.8 ± 0.4 m/s	MMSE: 26.7 ± 2.7
Pohl et al. (32)	Stroke	24 *(8)*	66.5 ± 9.1	46.3 ± 32.3 months	10MWT: 0.7 ± 0.3 m/s	MMSE: 28.6 ± 1.7
	Control	12 *(6)*	72.7 ± 8.0	–	10MWT: 1.0 ± 0.2 m/s	MMSE: 29.7 ± 0.7
Regnaux et al. (33)	Stroke	18 *(6)*	46.6 ± 12.3	13.7 ± 16.2 months	10MWT: 0.5 ± 0.3 m/s	“No major cognitive impairment”
	Control	10 *(3)*	25-55	–	*NM*	*NM*
Manaf et al. (30)	Stroke	30 *(13)*	55.0 ± 12.0	53.0 ± 73.0 months	10MWT: 0.9 ± 0.3 m/s	MMSE: 28.0 ± 2
Tisserand et al. (34)	Stroke	12 *(5)*	58.0 ± 12.8	27.0 ± 17.5 months	10MWT: 0.9 ± 0.4 m/s	MMSE: 29.0 ± 2.0
	Control	10 *(6)*	58.5 ± 4.0	–	10MWT: 1.5 ± 0.2 m/s	MMSE: 29.0 ± 0.3
Yang et al. (37)	Stroke	88 *(24)*	62.6 ± 7.8	105.9 ± 61.6 months	10MWT: 14.1 ± 6.9 s	MoCA: 24.8 ± 2.9
Yang et al. (36)	Stroke	61 *(15)*	62.9 ± 7.8	111.9 ± 66.7 months	10MWT: 14.5 ± 8.1 s	MoCA: 25.3 ± 2.4
	Control	32 *(14)*	61.0 ± 7.3	–	10MWT: 8.3 ± 1.5 s	MoCA: 26.6 ± 2.5

*6MWT, 6-min Walk Test; 10MWT, 10-meter Walking Test; CMSA, Chedoke-McMaster Stroke Assessment; DSST, Digit Symbol Substitution Test (correct responses in 90 s); m, meters; MMSE, Mini-Mental State Examination (max 30); MoCA, Montreal Cognitive Assessment (max 30); m/s; NM, Not mentioned; RBANS, Repeatable Battery for the assessment of Neuropsychological Status (correct responses in 90 s); SOMCT, Short Orientation, Memory, and Concentration Test (max 28); SPMSQ, Short Portable Cognitive Status Questionnaire (max 10); VSN +, participants with visuospatial neglect; VSN -, participants without visuospatial neglect*.

#### Locomotor Tasks Description and Outcomes

A diverse array of locomotor tasks was used in the selected studies including simple forward locomotion (25, 28, 31, 33–38, 40), walking with direction changes (24, 26, 27, 29, 30, 32, 38, 41, 42), and avoiding obstacles during walking (36, 39). One study used a challenging locomotor task consisting of a 10-m walking test combining obstacles, tandem-walking paths, and stepping onto targets (20).

To assess gait performance, most studies measured spatiotemporal parameters, such as walking speed (20, 27, 28, 30, 31, 34, 35, 39–41), walking time (26, 28, 30, 36–38, 42), cadence (25, 29, 32, 41), stride length (25, 28, 30, 41) and stride time (30, 33, 41). In the locomotor tasks involving obstacle avoidance, outcomes such as success rate, minimal distance between the obstacle and the participant or onset of avoidance strategy were used to quantify the performance (20, 39). Only two studies focused on kinematic or kinetic outcomes (24, 34).

#### Cognitive Tasks Description and Outcomes

Participants were instructed to perform various cognitive tasks while walking. The most frequently used cognitive tasks were mental tracking tasks (20, 24, 25, 27, 30, 31, 34–38, 41), discrimination and decision-making tasks (26, 31, 39, 40, 42) and verbal fluency tasks (29, 32, 34, 37, 38, 41). In terms of outcomes, accuracy was the most frequently reported variable to quantify cognitive performance in the aforementioned categories. In 3 studies using discrimination and decision-making tasks (26, 40, 42), reaction time or a composite score, combining accuracy and reaction time, characterized cognitive performance. Fluency, grammatical complexity or semantic were also used to assess verbal fluency tasks (29, 32, 41).

No specific instructions regarding task prioritization were given to the participants during DT condition, with the exception of Manaf et al. (30) in which participants were instructed to prioritize the cognitive task while walking.

### Locomotor and Cognitive DTI in Stroke Participants

Seventeen studies (20, 25–36, 39–42) out of 20 contributed to reach the first and second objectives of this systematic review, i.e., to examine cognitive-locomotor DTI magnitude and pattern while walking in people who sustained a stroke and explore the impact of the nature of the tasks on DTI (Table 3). Three studies that were not considered (24, 37, 38) due to the absence of statistical comparison between single- and dual-task conditions.

**Table 3:** Dual-task interference on locomotor and cognitive performances according to the nature of the tasks in stroke survivors.

**References**	**Locomotor Task**	**DTI on locomotor performance**	**Cognitive Task**	**DTI on cognitive performance**
**FORWARD WALKING**				
Al-Yahya et al. (25)	Treadmill	Stride length: **↓** Cadence: **↓**	Serial subtraction (-7)	Counting rate: **↓** Accuracy: **NS**
Feld et al. (40)	Overground	Walking speed: **↓**	Auditory Stroop Test	Accuracy: **↓** Reaction time: **NS**
Goh et al. (35)	Overground at comfortable and maximum walking speed	Comfortable walking speed: **↓** Maximum walking speed: **↓**	Serial subtraction (-3)	Accuracy: **NS** (for both speed)
Hyndman et al. (28)	Overground	Walking time: **↑** Walking speed: **↓** Stride length: **↓**	Auditory mental task (shopping list)	Number of items correctly recalled items: **↓**
Patel and Bhatt (31)	Overground	Walking speed: **↓**	Visuomotor reaction time Serial subtraction (-2) Visual Stroop Test	***Visuomotor reaction time*** Reaction time: **↑** ***Serial subtraction (-2) & Visual Stroop Test*** Number of correct responses: **↓**
Regnaux et al. (33)	Treadmill	Stride time: **NS** Time of task: **NS**	Reaction time after an electrical stimulation	Reaction time: **↑**
Tisserand et al. (34)	Overground	Walking speed: **NS** Margin of stability width: **NS** Base of support width: **NS**	Counting forward and backward Semantic and phonemic verbal fluencies	Accuracy: **↓**
Yang et al. (36)	Overground	Walking time: **↑**	Serial subtraction (-3) Serial subtraction (-7)	Accuracy: **NS**
**WALKING WITH DIRECTION CHANGES**				
Chan and Tsang (26)	Back and forth (180°)	Turning time: **NS** Number of steps to turn: **NS** Walking time: **NS**	Auditory Stroop Test	Reaction time: **NS** Accuracy: **↓**
Denneman et al. (42)	Timed-Up-and Go	Walking time: **↑**	Auditory Stroop Test	Composite score: **↓**
Dennis et al. (27)	Oval walkway at preferred and maximum speed	***Serial subtraction (-3)*** Preferred walking speed: **↓** Fast walking speed: **↓** ***Clock Face Test*** Preferred walking speed: **NS** Fast walking speed: **NS**	Serial subtraction (-3) Clock face test	***Serial subtraction (-3)*** Accuracy: **NS** *(for both speed)* ***Clock Face Test** Preferred walking speed* Accuracy: **NS** *Fast walking speed* Accuracy: **↓**
Kemper et al. (29)	Oval walkway	Cadence: **NS** Time on task: **↓**	Spontaneous speech	Fluency: **↓** Grammatical complexity: **↓** Semantic content: **↓**
Manaf et al. (30)	Timed-Up-and Go	Walking time: **↑** Walking speed: **↓** Stride time: **↑** Stride length: **NS** Coefficient of variability of walking speed: **NS** Coefficient of variability of stride length: **NS** Coefficient of variability of stride time: **NS**	Serial subtraction (-3) (with prioritization)	Number of repeated trials: **↑** Number of correct responses: **↓**
Plummer-D'Amato et al. (41)	Oval walkway	***Auditory 1-back; Clock Face Test; Spontaneous speech*** Walking speed: **↓** Stride length: **↓** Cadence: **↓** Stride time variability: **NS** ***Clock Face Test; Spontaneous speech*** Stride time: **↓** ***Auditory 1-back*** Stride time: **NS**	Auditory 1-back Clock face test Spontaneous speech	***Auditory 1-back; Clock Face Test*** Reaction time: **NS** Accuracy: **NS** ***Spontaneous speech*** Utterances/narrative: **↓** Significant decline in words/narrative: **↓** Significant decline in pauses/utterance: **↓** Proportion of utterance with new information: **↓** Sentence length: **NS** Fillers/utterance: **NS** Sentence complexity: **NS** Proportion of grammatical sentence: **NS**
Pohl et al. (32)	Oval walkway	Cadence: **↓**	Spontaneous speech	Grammatical complexity: **↓** Semantic content: **↓** Speech rate: **NS** Fluency: **NS**
**WALKING WITH OBSTACLES**				
Aravind and Lamontagne (39)	Avoidance moving obstacle walking	Minimum absolute distance: Without visuospatial neglect: **↑** With visuospatial neglect: **↓** Delay of onset of avoidance strategy: Without visuospatial neglect: **NS** With visuospatial neglect: **↑** Walking speed: With visuospatial neglect: **↓** Rate of collision: Without visuospatial neglect: **NS** With visuospatial neglect: **↑**	Auditory Stroop Test: Word “cat” presented in high or low pitch (Cog-CAT) Words “high” or “low” presented in a high or low pitch (Cog-HL)	Cog-CAT accuracy: With and without visuospatial neglect: **↑** Cog-HL accuracy: With and without visuospatial neglect: **↑**
Yang et al. (36)	Crossing obstacle walking	Walking time: **↑**	Serial subtraction (-3) Serial subtraction (-7)	***Serial subtraction (-3)*** Accuracy: **↓** ***Serial subtraction (-7)*** Accuracy: **NS**
**OTHER WALKING TASKS**				
Timmermans et al. (20)	Challenging-physical walking task (three stepping walk, 2-m tandem-walking, 3 crossing-obstacles) Challenging-projected walking task (projected obstacles)	Walking speed: **↓** Walking adaptability performance score: **↓**	Serial subtraction (-3)	Accuracy: **↓**

*NS, non-significant. ↑, increase; ↓, decrease in outcome measure*.

### Influence of the Nature of Both Tasks on DTI

Included studies were categorized according to the nature of both locomotor and cognitive tasks and a total of 11 different combinations were tested (Table 4). DTI in one or both tasks was reported in all dual-task conditions, except one (27). DTI pattern seemed to vary according to the nature of both tasks. While a mutual interference was observed in most combinations of locomotor and cognitive tasks (20, 25, 28–32, 36, 39–42), 2 task combinations resulted in a cognitive-related motor interference (27, 35, 36, 41) and 5 others resulted in motor-related cognitive interference (26, 27, 33, 34). Only one study reported no dual-task interference (27). Due to the heterogeneity of the nature of both tasks used and outcomes used in the included studies, it is difficult to conclude on the impact of the nature of locomotor and cognitive tasks on DTI patterns. However, it is interesting to highlight that when participants were asked to avoid obstacles while walking (20, 36, 39), decrements in both cognitive and locomotor performances were systematically observed. When investigating the impact of different cognitive tasks, more variability was observed.

**Table 4:** Dual-task interference patterns in people who sustained a stroke according to the categorization of the nature of the locomotor and cognitive tasks.

**Locomotor tasks**	**Cognitive task**	**Mutual interference**	**Cognitive-related motor interference**	**Motor-related cognitive interference**	**No dual-task interference**
Forward walking	Mental tracking tasks (*n* = 5)	*n* = 2 (25, 31)	*n* = 2 (35, 36)	*n* = 1 (34)	*n* = 0
	Discrimination and decision -making tasks (*n* = 2)	*n* = 2 (31, 40)	*n* = 0	*n* = 0	*n* = 0
	Verbal fluency tasks (*n* = 1)	*n* = 0	*n* = 0	*n* = 1 (34)	*n* = 0
	Working memory task (*n* = 1)	*n* = 1 (28)	*n* = 0	*n* = 0	*n* = 0
	Reaction time task (*n* = 2)	*n* = 1 (31)	*n* = 0	*n* =1 (33)	*n* = 0
Walking with direction changes	Mental tracking tasks (*n* = 4)	*n* = 1 (30)	*n* = 3 (27, 41)	*n* = 1 (27)	*n* = 1 (27)
	Discrimination and decision -making tasks (*n* = 2)	*n* = 1 (42)	*n* = 0	*n* =1 (26)	*n* = 0
	Verbal fluency tasks (*n* = 3)	*n* = 3 (29, 32, 41)	*n* = 0	*n* = 0	*n* = 0
	Working memory task (*n* = 0)	–	–	–	–
	Reaction time task (*n* = 0)	–	–	–	–
Obstacle walking	Mental tracking tasks (*n* = 1)	*n* = 1 (36)	*n* = 0	*n* = 0	*n* = 0
	Discrimination and decision -making tasks (*n* = 1)	*n* = 1 (39)	*n* = 0	*n* = 0	*n* = 0
	Verbal fluency tasks (*n* = 0)	–	–	–	–
	Working memory task (*n* = 0)	–	–	–	–
	Reaction time task (*n* = 0)	–	–	–	–
Other challenging walking	Mental tracking tasks (*n* = 1)	*n* = 1 (20)	*n* = 0	*n* = 0	*n* = 0
	Discrimination and decision -making tasks (*n* = 0)	–	–	–	–
	Verbal fluency tasks (*n* = 0)	–	–	–	–
	Working memory task (*n* = 0)	–	–	–	–
	Reaction time task (*n* = 0)	–	–	–	–

### DTI Comparisons Between Stroke Survivors and Age-Matched Healthy Adults

Seven studies statistically compared DTI between stroke survivors and age-matched healthy adults (24–26, 28–30, 32). Baseline differences between groups were first identified to indicate potential confounding factors. No significant difference in age means between groups was observed. All studies using walking speed as an outcome to assess baseline locomotor function showed that stroke survivors walked slower than age-matched healthy participants (24–26, 28, 30, 32). Among the 5 studies comparing baseline cognitive function between groups, poorer cognitive function in stroke survivors was highlighted in 2 of them (26, 32).

Overall, most studies demonstrated that stroke participants had greater DTI in cognitive performance, locomotor performance or both compared to age-matched healthy participants while dual-tasking (Table 5). Both greater locomotor and cognitive DTI were noticed in 3 studies (24, 28, 29), while greater DTI in either the locomotor or cognitive task were observed in the 2 other studies (30, 32). No difference in DTI between the stroke and age-matched healthy participants were observed in the remaining 2 studies (25, 26).

**Table 5:** Comparison in dual-task interference between stroke and age-matched control groups.

**References**	**Locomotion task**	**Locomotor results**	**Cognitive task**	**Cognitive results**
**Differences in cognitive and locomotor DTI**				
Mori et al. (24)	Oval walkway	***T*****-Test** **Trunk linear accelerations** DTI stroke > DTI healthy control	Serial subtraction (-3)	***T*****-Test** **Correct response rate** DTI stroke = DTI healthy control **Mistake rate** DTI stroke > DTI healthy control
Kemper et al. (29)	Oval walkway	**Mixed-design ANOVA** **Cadence** DTI Stroke = DTI healthy control **Time on task** DTI Stroke > DTI healthy control	Spontaneous speech	According to the authors **Fluency**: DTI stroke > DTI healthy control **Grammatical complexity:** DTI stroke > DTI healthy control **Semantic content:** DTI stroke > DTI healthy control
Hyndman et al. (28)	Forward walking	**Mixed-design ANOVA** **Walking time** DTI Stroke > DTI healthy control **Walking speed** DTI Stroke = DTI healthy control **Stride length** DTI Stroke = DTI healthy control	Auditory memory	**Mixed-design ANOVA** DTI stroke > DTI healthy control
**Differences in locomotor DTI only**				
Manaf et al. (30)	Timed-Up-and Go	**Mixed-design ANOVA** **Walking time** DTI Stroke = DTI healthy control **Stride length** DTI Stroke = DTI healthy control **Walking speed** DTI stroke > DTI healthy control **Stride time** DTI stroke > DTI healthy control **Coefficient of variability of walking speed** DTI Stroke = DTI healthy control **Coefficient of variability of stride length** DTI Stroke = DTI healthy control **Coefficient of variability of stride time** DTI Stroke = DTI healthy control	Serial subtraction (-3)^*^	**Mixed-design ANOVA** **Number of repeated trials** DTI Stroke = DTI healthy control **Number of correct responses** DTI Stroke = DTI healthy control
**Differences in cognitive DTI only**				
Pohl et al. (32)	Oval walkway	***T*****-Test** **Cadence** DTI stroke = DTI healthy control	Spontaneous speech	***T*****-Test** **Rate speech** DTI stroke = DTI healthy control **Fluency** DTI stroke = DTI healthy control **Semantic content** DTI stroke = DTI healthy control **Grammatical complexity** DTI stroke > DTI healthy control
**No differences in DTI**				
Al-Yahya et al. (25)	Treadmill	**Mixed-design ANOVA** **Stride length** DTI Stroke = DTI healthy control **Cadence** DTI Stroke = DTI healthy control	Serial subtraction (-7)	**Mixed-design ANOVA** **Counting rate** DTI Stroke = DTI healthy control **Accuracy** DTI Stroke = DTI healthy control
Chan and Tsang (26)	Back and forth (180°)	***T*****-Test** **Turning time** DTI Stroke = DTI healthy control **Number of steps to turn** DTI Stroke = healthy control **Completion time** DTI Stroke = healthy control	Auditory stroop test	***T*****-Test** **Reaction time** DTI Stroke = DTI healthy control **Accuracy** DTI Stroke = DTI healthy control

* *Prioritization of the cognitive task was asked*.

Regarding DTI patterns in stroke participants, mutual interference was detected in 5 of the 6 studies in which statistical tests were performed between single and dual-task conditions in locomotor and cognitive performances in stroke participants (25, 28–30, 32). The remaining study noticed a motor-related cognitive interference only (26). When focusing on the DTI patterns of age-matched healthy adults in these studies, mutual interference was less commonly observed than in stroke survivors. Indeed, results of these studies were equally distributed among the following categories: mutual interference (25, 30), motor-related cognitive interference (26, 29), and cognitive-related motor interference (28, 32).

## Discussion

Results from included studies showed that performing an additional cognitive task while walking has a detrimental impact on locomotor or/and cognitive performances in persons with stroke. Despite highly variable combinations of cognitive and locomotor tasks, DTI patterns seemed to vary depending on the nature of the tasks performed simultaneously. For the comparison of DTI magnitude and pattern between stroke and age-matched healthy participants, most of the studies observed greater locomotor and cognitive DTI in stroke survivors. In addition, it was more frequent to observe mutual interference in stroke survivors than in age-matched healthy participants.

### Cognitive-Locomotor DTI in Stroke Survivors and the Influence of the Nature of Both Tasks

The systematic review of 20 studies have shown that persons with stroke are likely to present DTI in one or both performances while simultaneously performing cognitive and locomotor tasks. These findings are largely consistent with those of Plummer et al. (10), in which 7 studies were included. In the latter scoping review, DTI pattern appeared to be more frequently cognitive-related motor interference, with some task combinations producing mutual interference, which slightly differs from our results in which mutual interference was clearly the most common pattern. A potential explanation for this discrepancy relates to the nature and/or complexity of the studied locomotor tasks. In Plummer et al. (10), none of the 7 included studies assessed DTI in challenging walking conditions. On the opposite, 3 studies included in the present systematic review used locomotor tasks with obstacle avoidance (20, 35, 39) and mutual interference was reported in all of these studies. Results were much more variable in obstacle-free walking tasks. It has been previously shown that a disproportionate amount of attention is required when people with stroke are walking and negotiating obstacles (43). From a mechanistic perspective, persons with stroke exhibit greater activity in prefrontal cortex, quantified by fNIRS, than young and elderly participants, when avoiding obstacles while walking (44). These results are in line with the *Central Capacity Sharing Model* making the assertion that DTI origins from limited processing resources (13). Indeed, mutual DTI pattern observed when persons with stroke are avoiding obstacles while walking reflected that both tasks must share the available attentional capacities (13, 14). Considering that the ability to negotiate obstacles is one of the environmental demands associated with community mobility (45), individuals with stroke, even the well-recovered, need to be informed and aware of the high risk of interference in both locomotor and cognitive performances when dual-tasking in their daily activities.

From another perspective, the nature of the cognitive tasks may also have an impact on DTI (31, 36, 46), depending on the specific cognitive functions required to execute them. Indeed, a meta-analysis suggests that cognitive tasks involving internal interfering factors (e.g., mental tracking) seem to disturb walking speed and cadence more than those involving external interfering factors (e.g., reaction time). It has been hypothesized that cognitive tasks involving internal interfering factors share more complex neural networks with gait control than those that involve external interfering factors (15). Yang et al. (36) also investigated the influence of cognitive task complexity on DTI, but in this case using a mental tracking task (serial-3 subtraction vs. serial-7 subtraction). In this study, stroke survivors, as well as age-matched healthy older adults, reduced their walking speed and answered with less accuracy while performing serial-7 subtractions than serial-3 subtractions. Taken together, these results suggest that the nature of the cognitive tasks involved in dual-tasking has an impact on the magnitude of locomotor and cognitive DTI.

Findings of the present systematic review related to the influence of the nature of the tasks on DTI emphasized the importance of assessing dual-task capacities with cognitive and locomotor tasks that are representative of community ambulation in everyday life.

### Difference in DTI Between Stroke Survivors and Age-Matched Healthy Adults

The present systematic review highlighted that individuals who sustained a stroke present greater DTI than age-matched healthy people. Several personal factors could explain this difference. Age can be ruled out as an explanatory factor in the present study. Although reputedly known to impact DTI (18, 47), age means were similar between stroke and healthy controls in the studies reviewed herein.

Between-group difference may be due to locomotor impairments, however. After a stroke, reduction in walking speed, as well as temporal and spatial-limb asymmetries are often observed (48). A significant negative correlation between comfortable walking speed (single-task) and locomotor DTI in elderly persons (46, 49), individuals with Parkinson's disease (17), multiple sclerosis (50) or stroke (51) was documented in previous studies. Overall, individuals with a slower comfortable walking speed demonstrate greater locomotor DTI. Based on the present review, differences in DTI between stroke survivors and age-matched healthy adults cannot be systematically explained by a difference in baseline locomotor performance, however. One of the included studies obtained between-group differences in locomotor and cognitive DTI while stroke and healthy participants walked with similar baseline cadence (29). On the opposite, no between-group difference in locomotor and cognitive DTI was highlighted when stroke and healthy participants showed difference in baseline locomotor functions (25, 26).

Cognitive functions such as attention, language, short-term memory and executive functions can also be impaired after a stroke (4). DT ability may be affected by cognitive deficits, as demonstrated in studies including persons with mild cognitive impairment and Alzheimer's disease (52–54). Even without impaired walking function (single task), older adults with cognitive impairment had greater locomotor DTI than elderly without any cognitive deficit (52, 54). Positive correlations between cognitive functions, such as processing speed (50, 55), short-term memory or sustained attention (55) and DT walking speed have been previously demonstrated in healthy older adults (55) and in people living with MS (50). In the present systematic review, most stroke survivors did not present any cognitive impairments (Table 2). Significant between-group differences in baseline cognitive function were identified in two studies (26, 32), however. In Chan et al. (26), no DTI difference was observed between stroke survivors and age-matched healthy adults, despite the presence of a baseline cognitive difference across groups. These findings suggest that different baseline cognitive function did not necessarily result in different cognitive DTI between stroke survivors and healthy controls.

In most studies, cognitive functions were assessed with global screening assessments, such as the Montreal Cognitive Assessment [MoCA; (56)] or the Mini-Mental State examination [MMSE; (57)] in order to identify participants with cognitive impairments prior to the experiments. It is likely that these screening tools are not sufficient to characterize specifically each cognitive domain that may be involved in dual tasking, such as attention or executive functions. For a better understanding of the relationships between cognitive function and DTI in stroke survivors, future studies should consider using a neuropsychological test battery.

## Limitations, Strengths, and Future Directions

We examined exclusively the studies reporting both locomotor and cognitive DTI in persons with stroke, similarly to the selection strategy used in Plummer et al. (10). Due to this rigorous criterion, approximately half of the full texts read were excluded because of missing information about cognitive DTI. Wajda et al. (16) observed the same limitation in studies focusing on DTI in persons living with multiple sclerosis. We strongly believe that adding this selection criterion has strengthened the interpretation of results.

A meta-analysis could have been performed but it was judged inadequate by the authors given the heterogeneity of task combinations used to assess dual-task abilities and outcomes used in the included studies. Using standardized dual-task paradigms and outcome measures in future studies would facilitate the synthesis of results through a meta-analysis.

## Conclusions

This systematic review demonstrated that persons who sustained a stroke are likely to present decrements in one or both performances (locomotor and cognitive) while walking and performing a cognitive task, simultaneously. The nature of the tasks (locomotor and cognitive) seemed to have an impact on DTI. Mutual interference was systematically observed when more challenging walking tasks involving obstacle avoidance were performed. Given this result, people who sustained a stroke are more likely to present interference in both cognitive and locomotor performances when walking in the community. Further studies are needed to strengthen this conclusion. In addition to highlighting the extent to which DTI is greater in stroke survivors than in age-matched healthy adults, the present study showed that individuals with a stroke present mutual interference more frequently than age-matched healthy adults. Baseline locomotor and cognitive functions cannot systematically explain difference in locomotor and cognitive DTI between these two populations.

## Data Availability Statement

The datasets presented in this article are not readily available because the present study reviewed published scientific papers. Data are available in these publications.

## Author Contributions

AD-B and AB: selected the studies and performed the methodological quality assessment. AD-B: extracted the data. AB: checked the accuracy of extraction. All authors: discussed the results and contributed to the final manuscript and contributed to the design of the study.

## Conflict of Interest

The authors declare that the research was conducted in the absence of any commercial or financial relationships that could be construed as a potential conflict of interest.
