# Supplementary material for: Cognitive-Locomotor Dual-Task Interference in Stroke Survivors and the Influence of the Tasks: A Systematic Review
Source: Front Neurol. 2020 Aug 18;11:882. doi: 10.3389/fneur.2020.00882 (PMC7461874; doi:10.3389/fneur.2020.00882)
Supplement: Supplementary file 1 [file Table_1.DOCX]

Supplementary Material

**MEDLINE via PubMed: Search strategy**

| #1 | stroke or strokes or ((cerebrovascular* or cerebral* or brain or brains) adj2 (accident or accidents or infarction or infarctions or ischemi*))).ti,ab. |
| --- | --- |
| #2 | exp stroke/ or stroke rehabilitation/ or brain ischemia/ |
| #3 | #1 or #2 |
| #4 | (gait or gaits or walk or walks or walked or walking or ambulat* or locomotion*).ti,ab. |
| #5 | exp walking/ |
| #6 | #4 or #5 |
| #7 | (((dual or simultane* or motor cognitive or cognitive motor) adj2 (task* or function* or performance* or interferenc* or interaction*)) or multitask* or divided attention).ti,ab. |
| #8 | multitasking behavior/ or exp psychomotor performance/ or exp neuropsychological tests/ |
| #9 | #7 or #8 |
| #10 | #3 and #6 and #9 |

**CINAHL: Search strategy**

| #1 | TI(stroke or strokes or ((cerebrovascular* or cerebral* or brain or brains) N1 (accident or accidents or infarction or infarctions or ischemi*))) or AB(stroke or strokes or ((cerebrovascular* or cerebral* or brain or brains) N1 (accident or accidents or infarction or infarctions or ischemi*))) |
| --- | --- |
| #2 | MH(Stroke+ or stroke patients or cerebral ischemia) |
| #3 | #1 or #2 |
| #4 | TI(gait or gaits or walk or walks or walked or walking or ambulat* or locomotion*) OR AB(gait or gaits or walk or walks or walked or walking or ambulat* or locomotion*) |
| #5 | MH(walking+) |
| #6 | #4 or #5 |
| #7 | TI(((dual or simultane* or motor cognitive or cognitive motor) N1 (task* or function* or performance* or interferenc* or interaction*)) or multitask* or divided attention) or AB(((dual or simultane* or motor cognitive or cognitive motor) N1 (task* or function* or performance* or interferenc* or interaction*)) or multitask* or divided attention) |
| #8 | MH(task performance and analysis+ or psychomotor performance+ or neuropsychological test) |
| #9 | #7 or #8 |
| #10 | #3 and #6 and #9 |

**EMBASE: Search strategy**

| #1 | stroke:ab,ti or strokes:ab,ti or (((cerebrovascular* or cerebral* or brain or brains) near/2 (accident or accidents or infarction or infarctions or ischemi*)):ab,ti) |
| --- | --- |
| #2 | 'Cerebrovascular accident'/exp OR 'brain ischemia'/de OR 'stroke rehabilitation'/de |
| #3 | #1 or #2 |
| #4 | Gait:ab,ti OR gaits:ab,ti OR walk:ab,ti OR walks:ab,ti OR walked:ab,ti OR walking:ab,ti OR ambulat*:ab,ti OR locomotion*:ab,ti |
| #5 | 'Walking'/exp |
| #6 | #4 or #5 |
| #7 | (((dual OR simultane* OR 'motor cognitive' OR 'cognitive motor') NEAR/2 (task* OR function* OR performance* OR interferenc* OR interaction*)):ti,ab) OR multitask*:ti,ab OR 'divided attention':ti,ab |
| #8 | 'Dual-task performance (test)'/exp OR 'psychomotor performance'/exp OR 'neuropsychological test'/exp |
| #9 | #7 or #8 |
| #10 | #3 and #6 and #9 |

**PEDro: search strategy**

| #1 | Dual-task + stroke |
| --- | --- |
| #2 | Dual-task + walk |
| #3 | Dual-task + gait |
| #4 | #1 and #2 and #3 |
